# HIN7/440: Evidence-based Consumer Health Information - The need for unbiased risk communication

**DOI:** 10.2196/jmir.1.suppl1.e38

**Published:** 1999-09-19

**Authors:** B Hoeldke, I Muehlhauser

**Affiliations:** ^1^University Hamburg, HamburgGermany

**Keywords:** Risk Communication, Mass-Screening, Evidence-based Healthcare, Informed Consent, Decision Making

## Abstract

Online consumer health information is rapidly growing. At the same time an active part of patients and consumers in decision making about preventive or therapeutic interventions is increasingly demanded. The basis for informed consumer choice is the communication of evidence-based scientific data in a format that is clearly understood by most lay persons. The way study results are presented influence decisions by health care providers and patients or consumers alike. The impact of framing of outcome data as either relative or absolute differences is well recognized. Outcome data should be reported as absolute numbers, absolute risk reductions or numbers needed to treat or to screen rather than as relative risk reductions. Beyond the question of whether relative or absolute differences are used, outcome data can be framed by either emphasising achievable benefits or the lack of such benefits. Presentation of data as the proportion of patients who remain free of a target outcome rather than the proportion of patients who benefit from a certain intervention could substantially influence decision making. So far, studies evaluating the communication of treatment results to patients were focussed on the benefits of the respective interventions. Such an approach is incompatible with unbiased informed decision making by the patient, client or consumer. In order to communicate outcome data in an objective manner the whole possible spectrum of data presentation should be considered. Both, the proportion of persons who are likely to benefit as well as the proportion of persons who are unlikely to benefit or likely to be harmed should be presented with equal emphasis. Instruments to judge the quality of printed or online consumer health information do not include rating the framing of outcome data (e.g. http:/www.discern.org.uk).In order to establish an online system of evidence-based consumer health information that provides unbiased evidence-based communication of outcome data mammography screening was used as a model. After screening the literature according to evidence-based medicine criteria the information on benefits and risks of mammography screening has been compiled. Results are communicated as simple self explaining illustrations as well as original numbers equally emphasising the various aspects of the outcome. In addition, unbiased information is provided on the test efficacy of mammography screening (false positive, false negative results), on other potential side effects or other beneficial effects of mammography screening such as the number of diagnostic surgical interventions following mammography or the psychological sequaele thereof, data on total mortality and precision or lack of precision of results. The described mammography screening consumer information system is being evaluated with experts and the target consumer population with the final goal of an online evidence-based consumer health information

